# ChoK-ing the Pathogenic Bacteria: Potential of Human Choline Kinase Inhibitors as Antimicrobial Agents

**DOI:** 10.1155/2020/1823485

**Published:** 2020-07-09

**Authors:** Moad Khalifa, Ling Ling Few, Wei Cun See Too

**Affiliations:** School of Health Sciences, Health Campus, Universiti Sains Malaysia, 16150 Kubang Kerian, Kelantan, Malaysia

## Abstract

Novel antimicrobial agents are crucial to combat antibiotic resistance in pathogenic bacteria. Choline kinase (ChoK) in bacteria catalyzes the synthesis of phosphorylcholine, which is subsequently incorporated into the cell wall or outer membrane. In certain species of bacteria, phosphorylcholine is also used to synthesize membrane phosphatidylcholine. Numerous human ChoK inhibitors (ChoKIs) have been synthesized and tested for anticancer properties. Inhibition of *S*. *pneumoniae* ChoK by human ChoKIs showed a promising effect by distorting the cell wall and retarded the growth of this pathogen. Comparison of amino acid sequences at the catalytic sites of putative choline kinases from pathogenic bacteria and human enzymes revealed striking sequence conservation that supports the potential application of currently available ChoKIs for inhibiting bacterial enzymes. We also propose the combined use of ChoKIs and nanoparticles for targeted delivery to the pathogen while shielding the human host from any possible side effects of the inhibitors. More research should focus on the verification of putative bacterial ChoK activities and the characterization of ChoKIs with active enzymes. In conclusion, the presence of ChoK in a wide range of pathogenic bacteria and the distinct function of this enzyme has made it an attractive drug target. This review highlighted the possibility of “choking” bacterial ChoKs by using human ChoKIs.

## 1. Introduction

Alongside the revolutionary development of antibiotics, many bacteria adapted swiftly and developed resistance to it. Nowadays, there are no efficient ways to combat the dreadful diseases caused by these adapted bacteria. A novel solution is needed to control these diseases. More so, in this era, that is denoted by the Centres for Disease Control and Prevention (CDC) as a “post-antibiotic era” [1]. In which the development of antibiotics has been halted and the “golden era” of antibiotic development has long passed [2], particularly when resistance has developed for every antibiotic that has been introduced [3]. This predicament has emerged since researchers have not been able to keep up with the emergence of antibiotic resistance or antimicrobial resistance (AMR) [4]. This has sped up the process of AMR development, due to the continuous use of the existing antibiotics [5–7]. Therefore, AMR must be dealt with immediately.

**Figure 1:**
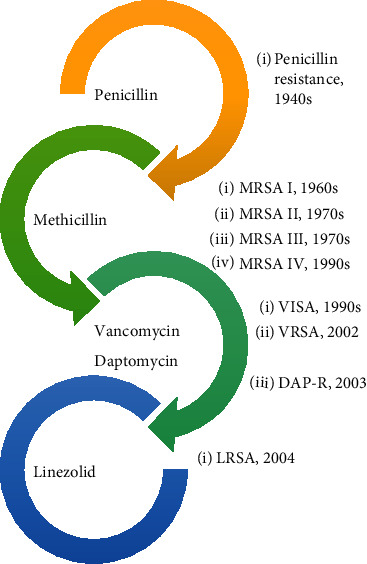
Trends in *S. aureus* treatment and resistance development.

There is no better example of bacterial resistance to antibiotics than *Staphylococcus aureus* (Figure 1). The eruption of the “antibiotic era” marked the AMR development of *S. aureus* towards penicillin after being developed in the 1940s [8]. This penicillin-resistant strain is known as the phage-type 80/81 strain and caused a pandemic [9]. It vaguely disappeared soon after the introduction of methicillin in 1960 [10]. Shortly after that, methicillin-resistant *S. aureus* type I (MRSA-I) has emerged and continued until the 1970s [11, 12]. Afterward, in the mid to late 1970s, MRSA-II and MRSA-III have emerged too, flagging MRSA as a pandemic, and then the 1990s marked the emergence of the smaller more mobile MRSA-IV [11].

The last line of effective treatment against MRSA is vancomycin (VAN) and daptomycin (DAP) [13]. Linezolid is still used as an alternative for vancomycin in treating MRSA [14]. Despite the discovery of those antibiotics, *S. aureus* has proven to be resilient. It developed AMR against vancomycin known as vancomycin-intermediate *S. aureus* (VISA) in the 1990s, vancomycin-resistant *S. aureus* (VRSA) in 2002 [11], and daptomycin in 2003 (DAP-R) [15]. To combat VISA and VRSA, daptomycin [16] and linezolid [17] are used, but resistance to linezolid has also developed in 2004 alongside DAP-R [14]. The quick adaptation of *S. aureus* and many other bacteria that developed AMR needs a novel solution that puts humanity as the front runner of the race once again.

The bacteria have proven to be adept in deploying survival tactics to resist antibiotics, not just *S. aureus* but also *Haemophilus influenzae* [18, 19], *Neisseria meningitidis* [20, 21], *Streptococcus pneumoniae* [22–24], *Streptococcus mitis* and *Streptococcus oralis* [25–27], and many others. The strategies of AMR include (i) the alteration of the antibiotic molecule itself via destruction or inactivation, (ii) limiting the antibiotic inside the cell via reduced penetration or the expression of efflux pumps that excrete the antibiotic outside the cell, and (iii) target site alteration via a mutation in the gene, enzymatic reaction, or replacing the target site with a new site unaffected by the antibiotic [28]. Therefore, antibiotic resistance is considered a virulence factor for the bacteria as it enables a pathogen to survive not only the host defense mechanism but also drugs used to treat the infection [29].

These strategies of the microbial resistance against antibiotics are the reason behind the incredible speed of AMR development. However, other community-based reasons are behind it as well, most notably, self-medication from previously prescribed drugs [30]. The availability of antibiotics over the counter in developing countries is another major factor in AMR development [31]. These facts urge the need for increasing public awareness and developing a novel method to combat AMR. Perhaps, the key would be the usage of novel drugs designed for eukaryotic cells that have proven to be effective in prokaryotic cells, nanoparticles (NPs), that hold the potential to solve the riddle of AMR, or even a combination between those two solutions to produce a highly effective antibiotic. This might even hinder the ability of the bacteria to develop AMR against it.

One of the promising novel drugs that have the potential to be an effective antimicrobial agent is the choline kinase (ChoK) inhibitors (ChoKIs). ChoK is already an established drug target in eukaryotes [32] and this enzyme has been found to exist in most species including some prokaryotes [33]. In humans, ChoK is the first enzyme in the CDP-choline pathway for the synthesis of phosphatidylcholine (PC), the main component of the membrane lipid bilayer. PC is also synthesized by an alternative phosphatidylethanolamine-N-methyltransferase (PEMT) pathway, which occurs almost exclusively in the liver [34]. Human ChoK (hChoK) exists in alpha and beta isoforms, and the upregulation of especially hChoK alpha has been implicated in various cancers; therefore, many ChoKIs have been designed and synthesized to specifically inhibit the alpha isoform as anticancer agents [35]. The first ChoKI was a dicationic choline-mimetic hemicholinium-3 (HC-3) that was able to decrease the level of phosphorylcholine and inhibit growth factor-induced DNA synthesis *in vitro* [36]. However, HC-3 also produces toxic side effects by inhibiting high-affinity choline transporters and acetylcholinesterase [37]. As the potential of ChoK inhibition in the anticancer strategy became more obvious, more ChoKIs were synthesized based on HC-3 as a prototype. The first-generation HC-3 derivatives were bis-pyridiniums with MN58b as the most potent followed by second-generation bis-quinoliniums including RSM-932A or TCD-717, which showed even better antiproliferative properties and have completed phase I clinical trials [38, 39]. Following the same pattern, more ChoKIs including EB-3D and EB-3P were synthesized for reduced toxicity and increased solubility [40]. Using *in silico* screening, a compound termed CK37 was found to inhibit hChoK alpha and selectively suppressed the growth of neoplastic cells [41]. The focused library screening had also identified another hChoK inhibitor, CCIC-0019 that is equipotent to MN58b and >500 times more potent than CK37 [42]. Importantly, ChoK inhibition only shows an antiproliferative effect on cancer cells but not on normal cells [39]. Besides demonstrating a promising effect on cancer [39, 43], ChoKIs also showed potential in targeting parasites such as *P. falciparum* [44–48] and *Trypanosoma brucei* [49] as well as modulating autoimmune diseases towards the treatment of rheumatoid arthritis [50–52]. Recently, it was suggested that ChoKIs could be useful for the treatment of acute and chronic inflammation-related diseases as they were effective in animal models [53]. In prokaryotes, ChoK activity has been confirmed in *S*. *pneumoniae* [54] while many other bacterial pathogens possess a putative ChoK gene, such as *S. aureus*, *Bacillus subtilis*, *Clostridium perfringens*, and *Clostridium botulinum* [35]. This significantly indicates the possibility of using eukaryotic ChoKIs on a wide range of pathogenic bacteria especially those that have developed AMR to currently available antibiotics.

## 2. Bacterial Membrane Lipids

Lipid synthesis in bacteria is a diverse and complex process that produces many lipids that form the bacterial membranes, including phosphatidylcholine (PC), phosphatidylethanolamine (PE), phosphatidylserine (PS), phosphatidylinositol (PI), phosphatidic acid (PA), phosphatidylglycerol (PG), cardiolipin (CL), lysyl-phosphatidylglycerol (LPG), glycolipids (GLs), and diacylglycerol (DAG) [54]. Gram-positive bacteria possess a thick murein cell wall and a cytoplasmatic membrane, while Gram-negative bacteria possess outer and inner membranes, and between them exists a thin murein cell wall [55–57]. The outer membrane consists mainly of lipopolysaccharide (LPS) [58]. Lipid A, in particular, forms the outer leaflet [59] and mediates virulence [60]. Lipid A is a vital component and a drug target in Gram-negative bacteria [61, 62]. *S. pneumoniae* survival and infection depend on the cell wall [63]. Choline (Cho) is essential for the cell wall [64]. Phosphorylcholine (ChoP) is synthesized from Cho by ChoK [65, 66]. ChoP involvement in the production of teichoic acids (TA) in the form of lipoteichoic acid (LTA) attached to the membrane and cell wall teichoic acid (CTA) indicates that ChoP is also essential [65, 67, 68]. ChoP is also involved in the synthesis of type IV LTA of *S. mitis* and *S. oralis* [69, 70]. LTA is an essential virulence factor and a potential drug target as it facilitates resistance to beta-lactams [71].

LPS is an important virulence factor [72], besides being an essential component. LPS can cause endotoxic shock, articulate the protective barrier of the outer membrane [55], and sensitize the host immune system [57]. It is due to the critical addition of ChoP to LPS and TA [73], even though this modification is considered rare [74]. ChoP modification aids survival of bacteria in the host [75, 76] and even nematodes [77], assists in recognition by host immunity [78] and adhesion facilitation [78, 79], promotes colonization [75, 76, 80], acts as an attachment to surface protein ligands and bacteriophage anchor [81], and reduces genetic alteration and bacterial autolysis [82].

PC is the most abundant phospholipid in eukaryotic cells, and it is estimated to be present in 10–15% of all bacteria [83, 84]. PC in bacteria acts as an intermediate for the biosynthesis of diacylglycerol-based phosphorus-free membrane lipids [84]. PC facilitates bilayer formation [83], proper membrane protein folding [85], and survival during different environmental changes [83], decreases susceptibility to antibiotics targeting bacterial membranes [75], and is critical in the microbe-host interactions [86]. Many bacteria showed diminished virulence in PC-deficient states such as *Legionella* sp. [87], *Brucella abortus* [88], and *Agrobacterium tumefaciens* [89]. In contrast, *Pseudomonas aeruginosa* showed no change in virulence in the PC-deficient state [90]. Since virulence factors should be considered in the process of selecting a novel drug target [91], ChoK would meet this criterion considering the importance of ChoP and PC in virulence.

## 3. ChoK in Bacterial Lipid Biosynthetic Pathways

**Figure 2:**
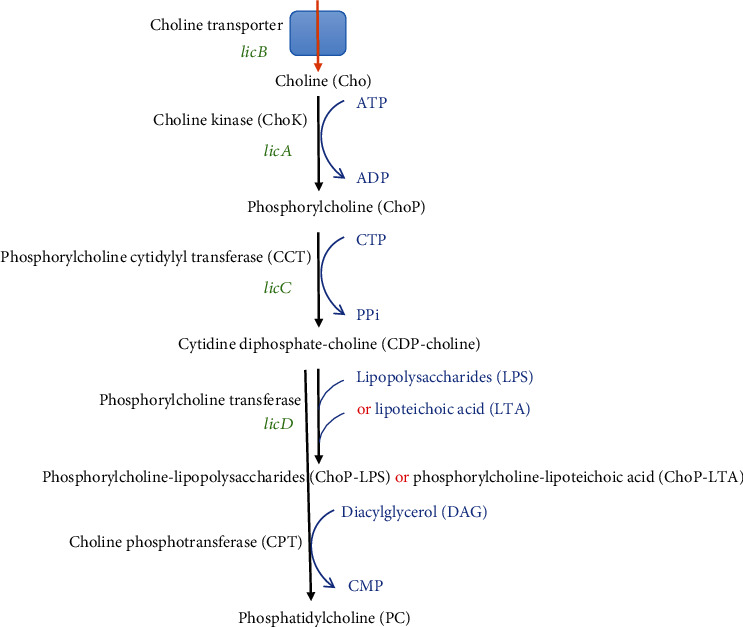
Pathways involving ChoK in the bacterial synthesis of ChoP-LPS, ChoP-LTA, and PC. The genes encoding for the responsible enzymes are in green and substrates are in blue.

As shown in Figure 2, ChoK (*licA*) is responsible for the phosphorylation of choline by using adenosine triphosphate (ATP) to ChoP in the cytoplasm of bacteria [35, 65, 92]. The uptake of extracellular choline is via the choline transporter (*licB*) [67, 74, 93]. Afterward, ChoP is activated into CDP-choline in the cytoplasm by phosphorylcholine cytidylyl transferase (*licC*) utilizing cytidine triphosphate (CTP) [35, 92]. ChoP is then transferred from CDP-choline by phosphorylcholine transferases such as *licD* in Gram-negative *H. influenzae* to LPS or *licD1* and *licD2* in Gram-positive *S. pneumoniae* to preteichoic acid for the synthesis of TA [84, 94, 95]. Lastly, TA is incorporated into the cell wall and membrane by teichoic acid flippase (*tacF*) [96]. In eukaryotes, the CDP-choline is used to synthesize PC by choline phosphotransferase (CPT); a similar step has recently been reported in *Treponema denticola*, which is catalyzed by 1,2-diacylglycerol choline phosphotransferase [97]. It is worth noting that although PC could be found in only about 15% of all bacteria [84] and ChoP produced by ChoK (*licA* gene product) might be rare [74], the *licA* gene homologs have been predicted in a number of pathogenic bacteria. Further research to confirm the activities of these putative ChoKs is required to realize the potential of using ChoKIs to fight against infections caused by more diverse species of bacteria.

**Figure 3:**
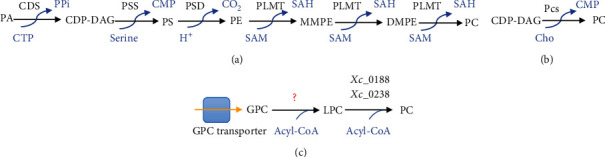
Phosphatidylcholine biosynthetic pathways in bacteria other than the CDP-choline pathway: (a) PE methylation pathway; (b) Pcs pathway; (c) GPC pathway. Abbreviations not described in the text: PPi: inorganic pyrophosphate; CMP: cytidine monophosphate; SAH: S-adenosylhomocysteine.

Since PC synthesis by the CDP-choline pathway is unique for certain species of *Treponema* [97], it is important to know the other pathways for PC synthesis that have been reported in prokaryotes. There are three other pathways for PC synthesis in bacteria (Figures 3(a)–3(c)) [54, 84, 86]. The PE methylation pathway (Figure 3(a)) [54, 83], where PC is synthesized from PE in a sequence of three steps catalyzed by phospholipid *N*-methyltransferase (PLMT) with methyl donor *S*-adenosylmethionine (SAM) to form monomethylphosphatidylethanolamine (MMPE), dimethylphosphatidylethanolamine (DMPE), and then PC [54]. MMPE is the last product in a few bacteria such as *Xanthomonas campestris* [98]. PE is produced from CDP-DAG by condensing it with serine at first by phosphatidylserine synthase (PSS) to form PS, which is decarboxylated to PE by phosphatidylserine decarboxylase (PSD) [54]. CDP-DAG on the other hand is produced from PA by CDP-DAG synthase [54] Secondly, the phosphatidylcholine synthase (Pcs) pathway (Figure 3(b)) [99] is comprised of a single step of condensing CDP-DAG with Cho by the Pcs enzyme [54, 99]. Finally, the glycerophosphorylcholine (GPC) pathway (Figure 3(c)) [54, 86] was reported only in *X. campestris* [98], *S. mitis*, and *S. oralis* [86]. In *X*. *campestris*, extracellular GPC is transported and converted via two-step acyl-CoA-dependent acylation to lysophosphatidylcholine (LPC) by unknown enzymes and then to PC by acyltransferases *Xc*_0188 and *Xc*_0238 [98]. Besides the abovementioned pathways, PC is also obtained via acylation of lysophospholipids [100]. How these alternative PC and other phospholipids biosynthetic pathways react, during inhibition of ChoK in bacteria to sustain growth requires further investigation to fully assess the potential of ChoKIs as antimicrobial agents.

## 4. Eukaryotic ChoKIs on Parasites

Unlike bacteria, the use of eukaryotic ChoKIs on parasites has gained more attention. One of the most interesting examples is the malarial parasite, *P. falciparum* that has developed chloroquine resistance and urgently needs novel drugs to control the disease [44]. ChoK is the first enzyme in the CDP-choline pathway for the synthesis of PC, the major (40 to 50% of total phospholipids), and the essential phospholipid in the membrane of *P*. *falciparum* [45, 48]. Inhibition of *de novo* PC synthesis by choline analog has been suggested as realistic malaria therapy even against pharmacoresistant strain [101]. Thus, inhibition of *P*. *falciparum* ChoK (*Pf*ChoK) was regarded as a potential antimalarial strategy. It was found that the primary sequence and tertiary structure of *Pf*ChoK catalytic sites are conserved compared with other ChoKs [44]. *Pf*ChoK is expressed at a higher level during growth phases of this parasite [47] and it becomes less viable after ChoK inhibition [48]. Second-generation (MN58b) and third-generation (RSM-932A) ChoKIs with minimal toxicity to humans have been shown to inhibit *Pf*ChoK and led to reduced parasitemia by impairing the maturation and invasion of *P. falciparum* [44]. MN-58b and RSM-932A demonstrated the potent antiplasmodial effect by inhibiting *Pf*ChoK in the low nanomolar range [44] possibly due to high sequence identity (~69%, when only conserved residues of ATP and Cho binding sites are considered) at the active sites compared to hChoK [45].

Other parasites utilizing the CDP-choline pathway for the synthesis of PC and carrying the ChoK gene are also potential targets for ChoKIs, most notably *Toxoplasma gondii*, which exhibits drug resistance [102], plasticity of biomass creation and gene expression [103], and nucleotide and central carbon metabolism resilience [104, 105]. Disruption of the CDP-choline pathway for PC synthesis and membrane biogenesis by pharmacological inhibition of *T. gondii* ChoK has been proposed as an effective way to arrest the growth of this organism [103].

*Entamoeba histolytica* is another deadly parasite that exhibits drug resistance with confirmed gene encoding active ChoK [106]. Therefore, *Eh*ChoK can also be tested for inhibition by ChoKIs. It must be noted that nematode parasites have also demonstrated other pathways of PC synthesis [107]. One such example is *Leishmania major*, in which despite having ChoK in the CDP-choline pathway for PC synthesis, this parasite has shown sustained growth in phosphorylcholine cytidylyltransferase- (CCT-) deficient state, which suggests the utilization of other alternative pathways of PC synthesis [108].

## 5. Eukaryotic ChoKIs Have the Potential to Be Prokaryotic ChoKIs

The use of eukaryotic ChoKIs for the inhibition of bacterial growth was first demonstrated by HC-3 inhibition of Gram-positive *S*. *pneumoniae* ChoK (*Sp*ChoK) [35]. Subsequently, two more eukaryotic ChoKIs, the MN-58b and RSM-932A, both are more potent inhibitors of human hChoK, were found to exhibit several orders of magnitude stronger inhibition against *Sp*ChoK than HC-3 [92]. Based on these examples, it was suggested that many drugs used to control diseases in eukaryotes have the potential to be used against the same protein targets in prokaryotic pathogens, especially whenever it meets certain criteria including conserved primary and tertiary structures [92]. *Sp*ChoK showed conservation of tertiary structure and catalytic site residues with hChoK [63, 92] and that could be the reason for the established eukaryotic ChoKIs (previously tested as anticancer drugs) to effectively inhibit *Sp*ChoK activity and distort the cell wall of *S. pneumoniae* [35].

**Figure 4:**
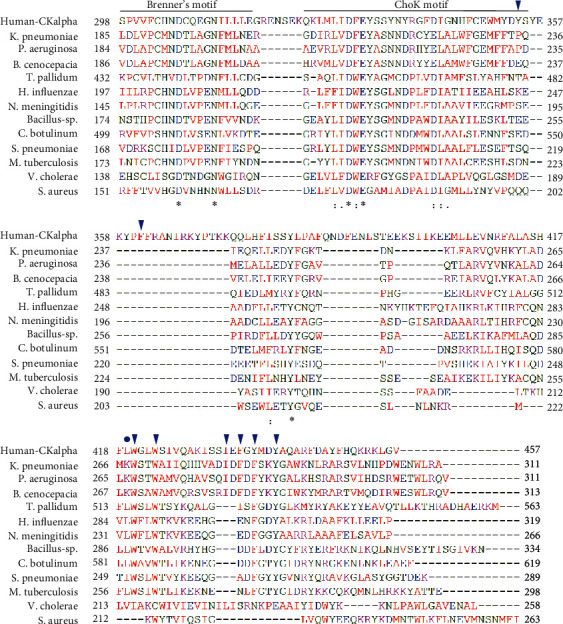
Amino acid sequence alignment of ChoK C-terminal from human and selected pathogenic bacteria. Sequence alignment was made by Clustal Omega available at https://www.ebi.ac.uk/Tools/msa/clustalo/ [111]. Asterisk, colon, and period indicate single fully conserved residue, conservation between groups of strongly similar properties, and conservation between groups of weakly similar properties, respectively. Inverted blue triangles indicate the hydrophobic residues forming interactions with HC-3 in hChoK alpha [110]. The blue circle indicates the hydrophobic residue that influences the plasticity of HC-3 binding groove. GenBank accession numbers for Human-CKalpha (NP001268.2), *K*. *pneumoniae* (PUG97579.1), *P*. *aeruginosa* (PTZ28970.1), *B*. *cenocepacia* (ODN63053.1), *T*. *pallidum* (APT97059.1), *H*. *influenzae* (AIB45944.1), *N*. *meningitidis* (SPY01484.1), *C*. *botulinum* (KON14313.1), *S*. *pneumoniae* (VTW72173.1), *Bacillus* sp. (AFS006103), *M*. *tuberculosis* (SGD50227.1), *V*. *cholerae* (QEO43700.1), and *S*. *aureus* (AXU08810.1) are shown.

The promising outcome from *Sp*ChoK inhibition highlights the potential of using existing ChoKIs for pathogenic microorganisms with ChoKs having high amino acid sequence homology with the human enzyme at the active sites.  Figure 4 shows the sequence alignment of the C-terminal of hChoK and putative ChoKs from some pathogenic bacteria showing conserved amino acids at the active sites. The conserved aspartate and asparagine in the Brenner's phosphotransferase motif have been shown to interact with the phosphate group of phosphorylcholine (product) in the solved structure of human ChoK (Asp-306 and Asn-311), and the aspartate residue is critical for catalytic activity [109]. In the ChoK motif, the conserved aspartate is important for coordinating magnesium ion for catalysis of the human enzyme (Asp-330) and mutation of this amino acid resulted in total loss activity [109]. Besides sequence conservation at the catalytic sites, conservation of amino acids previously identified to interact with ChoKI is also a crucial supporting evidence for the use of eukaryotic ChoKIs against bacteria ChoKs. Based on the crystal structure of hChoK alpha-HC-3 complex [110], several hydrophobic hChoK alpha residues interacting with HC-3 (inverted triangles in Figure 4) could also be found in bacteria ChoKs. Particularly, Trp-420, Trp-423, and Tyr-440 in hChoK alpha are also present in all the bacteria ChoKs except *V. cholerae* and *S. aureus*. Tyr-437 in hChoK was substituted with a different hydrophobic phenylalanine residue in the bacteria. Leu-419 (blue circle in Figure 4) of hChoK alpha that is neighbouring the active site and influences the plasticity of HC-3 binding groove was also relatively conserved (six out of twelve compared sequences) among bacterial ChoKs. Based on the sequence conservation of the active sites and some residues interacting with HC-3, most of these bacterial ChoKs, if confirmed to be active and expressed in the respective organisms, could be potential targets of human ChoKIs.

## 6. Bacteria with *lic* Operon and ChoK Gene

The study of the operon or the cluster of genes that usually code for proteins in the same metabolic pathway has been conducted to predict the presence of a certain pathway [84]. The *lic* operon responsible for the CDP-choline pathway remains studied in few bacteria only. These bacteria include *S. pneumoniae*, *S. mitis*, *S. oralis*, and *H. influenzae*.

In *S. pneumoniae*, the *lic* operon contains three regions [67]. *lic*1 encodes for Cho transporter, the ChoK, and phosphorylcholine cytidylyl transferase, which are expressed by *licB*, *licA*, and *licC* genes, respectively. The *lic1* region also contains *tarJ* gene encoding NADPH-dependent alcohol dehydrogenase for ribitol-phosphate synthesis and *tarI* gene encoding cytidylyl transferase for cytidine-diphosphate synthesis. *lic*2 region contains *licD1* and *licD2* genes that code for phosphorylcholine transferases and the *tacF* gene for teichoic acid flippase. The remaining *lic*3 region contains the *licD3*, *spr1221*, *spr1222*, *spr1223*, and *spr1224* genes [67].

*S. mitis* and *S. oralis* are closely related to *S. pneumoniae*. In *S. oralis*, *lic1* contains *licB*, *licA*, *licC*, *tarI*, and *tarJ* genes, while *lic4* contains *licd3*, *licd4*, *tacF*, *aroK*, *aroA*, *pheA*, and others such as *sor_0763* and *sor_0759*; however, in *S. mitis*, *lic1* contains *licB*, *licA*, *licC*, *tarI*, and *tarJ*; *lic2* contains *licD1*, *licD2*, and *tacF* genes; and *lic3* contains *licD3*, *aroK*, *aroA*, *pheA*, and others such as *smi0766* and *smi_0768* to *smi_0771* [67]. In *H. influenzae*, the *lic* operon contains three regions: *lic1* contains the *licB*, *licA*, *licC*, and *licD* genes; *lic2* encodes for Gala(1-4)bGal; *lic3* contains *galE* gene encoding for galactose epimerase [112]. Additionally, *H. influenzae* undergoes phase variation in the expression of LPS leading to a variety of oligosaccharide epitopes [113]. Interestingly, thousands of bacterial species have demonstrated to possess a putative ChoK gene [92]; confirmed ChoK activity has also been reported for Gram-negative *H. influenzae* and the Gram-positive *S. pneumoniae* [63]. These data indicate the potential existence of the ChoK gene, and that however different the organization of the *lic* operon is, it still encodes for the enzymes necessary for the CDP-choline pathway in different bacterial species. This demonstrates the need for more research in the confirmation of ChoK gene function and *lic* operon to combat pathogenic microbes by inhibiting ChoK activity.

It is interesting to note that the choline transporter (*licB* gene product) could also be a potential target for human ChoKIs like HC-3 and some of its derivatives that showed the undesired neuronal toxicity due to perturbation of choline transport into the cells. These inhibitors could be repurposed for choline uptake inhibition in pathogenic bacteria to achieve the same effect as inhibiting ChoK. Since there is no evidence for *de novo* synthesis of choline in prokaryotes and because choline uptake from exogenous sources is energetically more favorable than *de novo* synthesis, different uptake mechanisms for choline transport across the membrane have evolved in bacteria [114]. Inhibition of choline transporters can be expected to produce a detrimental effect on bacterial growth by blocking the supply of choline for the synthesis of cell wall and LPS components (as described above), glycine betaine (an important osmoprotectant) [115] or trimethylamine (produced by anaerobic degradation of choline and used as a carbon source or precursor to marine osmolyte or greenhouse gas, methane) [116].  Table 1 shows a list of pathogenic bacteria containing ChoKs and choline transporters. Based on this idea, choline uptake inhibitors developed for human neurons such as ML352 [117] could also be tested for blocking bacterial choline uptake system. More inhibitor candidates would certainly improve the outcome as bacteria, including *H. influenzae* might have multiple mechanisms for choline uptake other than *licB* transporter [93]. Besides choline transporters, another likely target of ChoKIs is the choline-binding proteins (CBPs) that play important roles in the viability and virulence of pneumococcus and related species. The CBPs have been proposed as targets for the design of new antimicrobials [91].

## 7. Nanoparticles as a Delivery Vehicle for ChoKIs

Nanoparticles (NPs) ranging from inorganic nanomachines to organic molecules like proteins, delivered as nanocapsules or nanospheres, are nanomedicines (as referred to by the National Institutes of Health) that are ushering a new era in disease diagnosis, treatment, and prevention [136]. NPs have a huge impact on the diagnosis of pathogens. In fact, if combined with theranostics, they would allow the combination of diagnosis, therapy, and monitoring in one platform [137]. According to Bobo et al. in the year 2016 [138], there were 51 nanomedicines approved by the USFDA, including the nanoformulations for antibacterial purposes. Various types of NPs used in the treatment of microbial infections have been discussed in a recent review by Lee et al. [139] and Yeh et al. [140]. NPs can be designed to carry antibiotics specifically to the pathogen at the site of infection. For example, to enhance antibiotic delivery to lung infection, NPs are incorporated into swellable microparticles for better deposition to deep lung [141]. At the site of infection, charge-based localization of NPs to the bacterial cell wall by using cationic peptide can be employed. More specific localization of NPs to the pathogen can be achieved by conjugating bacterium-specific ligand (small molecules, proteins, antibodies, or aptamers) to the NPs. Similarly, pathogens that invade and survive inside host cells can be specifically targeted by designing NPs that trigger uptake by the host cells [141]. In addition, surface charge-switching PLGA-PLH-PEG NPs were used to shield nontarget interaction at pH 7.4 and releasing the transported drug only in low pH caused by bacterial metabolism or host immune response, thus increasing the efficacy of antimicrobics [142]. The use of silver NPs could also enhance antibiotic activity against bacteria [143].

NPs attack bacteria via multiple mechanisms, mainly through oxidative stress [144], prevention of biofilm formation [145], and direct action on the cell wall [146]. However, when combined with an antibiotic, it will help restore the antimicrobial efficacy [147]. NPs can act solely and combat bacteria alone or act as a vehicle or delivery system for antibiotics [1, 148]. More importantly, NPs can deliver drugs to the site of action [136] with minimal side effects [148], have the localization of an ample amount of the drug to overcome drug resistance [149], and even have the potential to prevent AMR from occurring [150].

Using an efficient targeted system, CARG-conjugated vancomycin-loaded nanoparticles that specifically target *S. aureus* have managed to subdue infection with lower systemic dose to minimize any potential side effect [151]. Other examples of antibiotics delivery by NPs include nanoparticles chelated with amoxicillin or ampicillin against resistant strains of *E*. *coli*, ZnO nanoparticles complexed with ciprofloxacin against *S. aureus* and *E. coli*, rifampicin and thymopentin inside micro-nanoparticle hybrid to treat deep lung infections, and ciprofloxacin in nanoparticle-hydrogel hybrid material (NP-gel) to inhibit *E. coli* forming biofilm [141]. Formation of biofilms by pathogenic microorganisms has been directly linked to chronic infections with increased disease severity, and NPs have provided the perfect platform to combat bacteria in biofilms through direct interaction with the extracellular polymeric substances (EPS) [152, 153]. Despite the much lower toxicity of the later generations of ChoKIs [40] compared to HC-3, packaging and delivery of ChoKIs to the site of action or infectious agents would allow high local concentrations while minimizing systemic side effects to the human host. The high local concentration of antibiotic is important because less than minimum inhibitory concentration (MIC), especially in the intracellular compartment of infected host cells, could result in the development of drug resistance [154]. Based on the advantages and versatility of NPs, we hypothesize that NP delivery of ChoKIs to the site of infections could increase its efficacy and specificity while reducing potential side effects. Depending on the types of infections, NPs with bacterium-specific ligands, antibiofilm properties, or features for internalization into host cells for intracellular infections could be selected as vehicles for ChoKIs while shielding the effect of the inhibitors from any human ChoKs or other unspecific targets.

## 8. What the Future Holds (Research Perspectives)

Despite the progress made in understanding the biochemical pathways for the synthesis of bacterial phospholipids and the confirmation of ChoK activities in prokaryotes, many questions still need to be answered to fully adopt ChoKIs for antibacterial in solving the AMR problem. Is there an alternative or compensatory pathway alongside the CDP-choline pathway involving ChoK for the synthesis of PC in the target pathogenic bacteria? How potent are ChoKIs developed as anticancer agents when used on bacterial ChoKs? Will there be any side effects on the host? Yet, considering the widespread AMR in so many human pathogens, the desperate search for new antimicrobial agents including ChoKIs warrants more efforts to address the above questions. Some of the possible answers or solutions have been discussed in this review, and the efficacy of ChoKIs against ChoK from a specific microbe should be evaluated experimentally. Even if currently available ChoKIs do not show the desired potency against bacterial ChoKs, they can become the lead compounds for the synthesis of more effective antimicrobials. Ideally, a new generation of ChoKIs with high specificity and potency for bacterial ChoKs is generated to avoid any negative side effects on humans. More specific inhibitors are also important because human ChoKIs might not directly compete for the active sites with substrates as indicated by the mechanism of inhibition of *Pf*ChoK by HC-3, MN58 (not competitive), and RSM-932A (uncompetitive) [44]. It was even reported that TCD-717 (also known as RSM-932A) did not bind directly in the choline-binding pocket of hChoK [155]. Biochemical and structural characterizations of purified bacteria ChoKs with selected inhibitors showing antimicrobial effect are still necessary to elucidate their modes of action. The same problem of AMR could also happen to ChoKIs; this is most likely through the activation of the alternative/compensatory pathway(s). Therefore, the other pathways (which could be predicted by gene sequence analysis) that might be activated to bypass the pathway involving ChoK must not be ignored.

## 9. Conclusions

We support the idea of using eukaryotic ChoKIs to inhibit prokaryotic ChoKs to battle AMR in bacteria. ChoKIs have shown promising results on *S*. *pneumoniae* by disrupting cell wall integrity that subsequently inhibited cell growth. Many disease-causing bacteria that have developed AMR possess the putative ChoK gene with highly conserved amino acid sequences at the catalytic sites. This makes them become plausible targets for currently available ChoKIs to add to the arsenal against the threat of infection by antibiotic-resistant bacteria. While the focus should be on the pathogenic microorganisms especially the ones that have developed AMR, there could be plenty of other applications for ChoKIs such as a relatively safe pesticide for bacteria that damage crops, i.e., agricultural applications. Biochemical characterization of more putative bacterial ChoKs would be required to confirm their activities, and basic parameters for ChoKIs such as IC_50_ and LD_50_ have to be determined to find the optimum dose of treatment. Synthesis of suitable NPs to ferry ChoKIs to the pathogen and shielding the unwanted side effects of ChoKIs from the host is also paramount to the success of the whole idea. In conclusion, more research in bacterial ChoKs would unlock the vast potential of ChoKIs as a new generation of antimicrobials, hence “choking” the pathogenic bacteria.

## Figures and Tables

**Table 1:** List of pathogenic bacteria containing ChoK and choline transporter. The presence of these proteins in the bacteria was supported by related literature or representative GenBank accession numbers to the putative ChoKs.

Bacterium	Supporting literature or GenBank accession numbers	
	ChoK	Choline transporter
*Bacillus subtilis*	AFS006103	[118, 119]
*Burkholderia cenocepacia*	ODN63053.1	[120, 121]
*Clostridium botulinum*	KON14313.1	[122, 123]
*Haemophilus influenzae*	AIB45944.1	[93]
*Klebsiella pneumoniae*	PUG97579.1	[124, 125]
*Mycobacterium tuberculosis*	SGD50227.1	[126, 127]
*Neisseria gonorrhoeae*	[128, 129]	[128, 130]
*Neisseria meningitidis*	[128, 129]	[128, 130]
*Pseudomonas aeruginosa*	[128, 129]	[131]
*Staphylococcus aureus*	AXU08810.1	[132]
*Streptococcus mitis*	OOS15958.1	[67]
*Streptococcus pneumoniae*	[63]	[67, 74, 86]
*Treponema denticola*	[97, 133]	[97]
*Treponema pallidum*	[133]	[97]
*Vibrio cholerae*	QEO43700.1	[134, 135]
